# A prospective post-marketing observational study of abemaciclib in patients with HR+, HER2− breast cancer in China

**DOI:** 10.1093/oncolo/oyag013

**Published:** 2026-01-23

**Authors:** Quchang Ouyang, Peng Yuan, Jianxia Liu, Yuee Teng, Zhihua Li, Xuening Ji, Lina Liu, Mopei Wang, Liqun Zou, Ling Xu, Suisheng Yang, Zhenxin Zhu, Liu Yang, Jinnan Li, Qiang Liu

**Affiliations:** Hunan Cancer Hospital Changsha, 410013, China; National Cancer Center/National Clinical Research Center for Cancer/Cancer Hospital, Chinese Academy of Medical Sciences and Peking Union Medical College, Beijing 100021, China; The First Affiliated Hospital of Soochow University, Suzhou 215006, China; The First Affiliated Hospital of China Medical University, Shenyang 110001, China; Nanchang People’s Hospital, Nanchang 330025, China; Zhongshan Hospital Affiliated to Dalian University, Dalian 116001, China; Nanyang Second General Hospital, Nanyang 473004, China; Peking University Third Hospital, Beijing 100191, China; West China Hospital of Sichuan University, Chengdu 610041, China; Peking University First Hospital, Beijing 100034, China; Gansu Cancer Hospital, Lanzhou 730050, China; Eli Lilly and Company, Shanghai 200041, China; Eli Lilly and Company, Shanghai 200041, China; Eli Lilly and Company, Shanghai 200041, China; Sun Yat-sen Memorial Hospital, Sun Yat-sen University, Guangzhou 510120, China

**Keywords:** abemaciclib, real-world, early breast cancer, locally advanced or metastatic breast cancer, safety, effectiveness

## Abstract

**Background:**

Abemaciclib is approved for hormone-receptor positive (HR+), human epidermal growth factor receptor-2 negative (HER2−) locally advanced/metastatic breast cancer (ABC) and high-risk early BC (EBC) in China. This prospective observational study describes real-world abemaciclib safety and effectiveness among Chinese patients with HR+/HER2− EBC/ABC.

**Methods:**

Adults with HR+/HER2− EBC/ABC who received abemaciclib between Mar-2022 and Jan-2024 across 32 Chinese centers were enrolled. Primary objective was to evaluate treatment-emergent adverse event (TEAE) and serious AE (SAE) incidence within 24 weeks of treatment. Secondary objective was to describe Week 24 event-free survival (EFS).

**Results:**

Among 387 patients with EBC and 539 with ABC (median age: 50.0 and 55.0 years, respectively), 89.1% and 82.4% received 150 mg twice daily initially. Abemaciclib was combined with aromatase inhibitors in 95.6% (EBC) and 60.5% (ABC) patients, and fulvestrant in 38.0% (ABC). TEAEs occurred in 85.5% (EBC) and 81.8% (ABC) patients (commonly diarrhea, neutrophil count decreased, and white blood cell count decreased), and SAEs in 3.9% and 7.4%. AEs led to discontinuation in 4.4% (EBC) and 7.2% (ABC) patients. Diarrhea was the most common AE leading to discontinuation (2.1% [EBC] and 1.7% [ABC]). Most patients who discontinued treatment due to AEs had no dose modification, while most with dose reduction/interruption remained on abemaciclib. Week 24 EFS rates (95% CI) were 99.7% (97.6–100.0; EBC) and 85.1% (81.3–88.2; ABC).

**Conclusions:**

Real-world safety, tolerability, and effectiveness of abemaciclib in Chinese patients with HR+/HER2– EBC and ABC were consistent with clinical trials, with no new safety signals, supporting its positive real-world benefit-risk profile.

Implications for Practice This represents the largest prospective observational study of abemaciclib to date and the first real-world study of abemaciclib safety and effectiveness among patients with breast cancer in China. In line with clinical trials, abemaciclib has a positive benefit-risk profile for real-world treatment of hormone-receptor positive (HR+), human epidermal growth factor receptor-2 negative (HER2−) locally advanced/metastatic breast cancer (ABC) and high-risk early BC (EBC). Given the increasing incidence of breast cancer in China, the large number of patients enrolled without the stringent eligibility criteria of clinical trials may better represent its epidemiology, enabling clinicians to relate these results to real-world practice.

## Introduction

Breast cancer is the most frequently diagnosed cancer in women, accounting for one quarter of cancer cases, and is a major cause of cancer-related death among women worldwide.^1^ The incidence of breast cancer is increasing rapidly in China,^2^ with cases of breast cancer in female Chinese patients comprising more than 18% of cases globally in 2020.^3^ Patients with breast cancer in China are typically diagnosed with more advanced disease, with an estimated 21.0% of patients presenting with stage III disease and 7.7% with stage IV disease, compared with 12.4% and 4.9% of patients in the US, respectively.^4^ With an increasing burden of breast cancer in China,^3^ effective and well-tolerated treatment strategies are needed.

Approximately 70% of breast cancers are estrogen- and/or progesterone-receptor positive (hormone-receptor positive [HR+]).^5^^,^^6^ Estrogen-induced activation of cyclin D dysregulates signaling via cyclin-dependent kinase (CDK) 4 and 6,^7^ key regulators of the G1 cell cycle phase, promoting phosphorylation of the retinoblastoma protein (Rb) to drive tumor cell proliferation.^6^^,^^8^^,^^9^ CDK4/6 have thus emerged as important therapeutic targets for patients with HR+ breast cancer.^6^^,^^10^^,^^11^ Abemaciclib, an oral, continuously dosed CDK4/6 inhibitor, inhibits Rb phosphorylation, leading to tumor cell senescence and apoptosis.^7^^,^^12^

In the global phase 3 MONARCH 2 and 3 trials, patients with HR+, human epidermal growth factor receptor-2 negative (HER2−) locally advanced or metastatic breast cancer (ABC) receiving abemaciclib treatment in combination with endocrine therapy (ET) significantly improved the outcomes of patients over ET, with a generally well-tolerated safety profile.^13^^,^^14^ Results of the phase 3 MONARCH plus study, which evaluated the efficacy and safety of abemaciclib in postmenopausal women predominantly enrolled in China, were consistent with the MONARCH 2 and MONARCH 3 studies, with no new safety signals observed.^15^ Efficacy has also been reported with abemaciclib in combination with ET among patients with high-risk HR+/HER2− early breast cancer (EBC) based on results of the phase 3 monarchE study, with improvements in invasive-disease-free survival (IDFS) with abemaciclib plus ET versus ET alone that were sustained over time, and a generally well-tolerated safety profile.^16–18^ Results of these studies led to the NMPA approval of abemaciclib in combination with ET for treatment of HR+/HER2– ABC and high-risk EBC in China, making abemaciclib the first CDK4/6 inhibitor to be approved in China for the treatment of EBC.

This prospective, observational, post-marketing study aimed to describe the real-world safety and effectiveness profiles of abemaciclib among Chinese patients with HR+/HER2− EBC and ABC. Interim results of this study in the ABC cohort showed favorable real-world safety and tolerability profiles of abemaciclib, with comparatively lower incidences of adverse events (AEs), serious AEs (SAEs), and AEs leading to discontinuation compared with the MONARCH 2, MONARCH 3, and MONARCH plus trials.^19^ Here we report the primary outcome results of this study among patients with EBC and ABC.

## Methods

### Study design

This prospective, multicenter, observational study (NCT05267327) aimed to evaluate the safety of abemaciclib in routine clinical practice settings in China. Eligible patients with HR+/HER2− EBC or ABC were enrolled from March 2022 to January 2024 across 32 study sites. This study adhered to international ethics guidelines, including the Declaration of Helsinki.^20^ The study protocol was approved by an Independent Ethics Review Board prior to initiation, and all patients provided written informed consent to the release of their data.

The first follow-up visit occurred at 4 (±1) weeks after first dose of abemaciclib treatment for regular safety monitoring. The primary analysis reported here was planned after approximately 900 patients completed 24 (±4) weeks follow-up from the first dose of abemaciclib. The observation period for safety endpoints was from abemaciclib initiation to 24-week follow-up, abemaciclib discontinuation (additional 30 days follow-up needed), loss to follow-up, or death, whichever occurred first. Patients were followed up to 2 years for effectiveness endpoints after abemaciclib initiation.

### Participants

The study population included adult patients (≥18 years old at enrolment) diagnosed with HR+/HER2− breast cancer who had been prescribed abemaciclib treatment. Patients were excluded if they had received abemaciclib before study enrolment, were simultaneously participating in a different study that included a treatment intervention and/or an investigational drug, were pregnant or breastfeeding (or planned to be pregnant within the duration of the study), or were contraindicated for the use of abemaciclib according to the Chinese label. Definitions used for classification of disease status (EBC or ABC) are outlined in the Supplemental Methods (see online supplementary material).

### Assessments and endpoints

The primary objective was to estimate the incidence of AEs and SAEs among Chinese patients with breast cancer within 24 weeks of receiving abemaciclib. Safety variables included the event term, coded using the Medical Dictionary for Regulatory Activities (MedDRA) v23.1, severity graded using the National Cancer Institute Common Terminology Criteria for Adverse Events (CTCAE) v5.0, dose adjustment, and relationship to abemaciclib. AEs of special interest were defined as: neutropenia (including neutropenia and neutrophil count decreased), infections, diarrhea, hepatic events (alanine aminotransferase [ALT] increased and aspartate aminotransferase [AST] increased), venous thromboembolism, and interstitial lung disease (ILD)/pneumonitis.

Secondary endpoints included the EFS rates at Weeks 24, 52, and 104. EFS events were defined as local recurrence, new metastatic disease, progression of metastatic disease, secondary malignancy from treatment initiation, or death. Week 24 data are presented here, with follow-up ongoing. Secondary endpoints also included the disease control rate (DCR) and objective response rate (ORR) in the ABC cohort at Week 24, and OS rate at Weeks 52 and 104.

### Statistical analysis

Analyses were conducted among the overall population, stratified by disease setting (EBC or ABC). Descriptive statistics were used to summarize patient disposition and safety results. The safety analysis set (SAS) included all patients who took at least one dose of abemaciclib. Disease progression was captured but not aggregated into overall AE counts. The EFS rate and its 95% confidence interval (CI) was estimated using the Kaplan–Meier method. Patients free from EFS events were censored at the last visit date. EFS rates were estimated among patients who took at least one dose of abemaciclib, had a baseline and any postbaseline follow-up EFS data. EFS analysis of EBC cohort included patients who received abemaciclib in the adjuvant setting. Analyses were conducted using Statistical Analysis System software, v9.4 or higher.

### Subgroup analyses

AE incidence and effectiveness were evaluated among the following pre-specified subgroups: age (<65 years; ≥65 years), and combination therapy (abemaciclib + aromatase inhibitor; abemaciclib + fulvestrant). AE incidence was also evaluated in patients with baseline hepatic disease and renal impairment. Effectiveness was also evaluated in patients with or without dose reduction and by treatment line among patients with ABC.

## Results

### Participants

Between March 2022 and January 2024, 933 patients were included in the SAS, with 387 patients in the EBC cohort and 539 patients in the ABC cohort; seven patients had unknown disease status (Figure S1—see online supplementary material for a color version of this figure). Baseline demographics and disease characteristics are summarized in Table 1.

**Table 1.** Baseline demographics and disease characteristics.

	EBC cohort (*N* = 387)	ABC cohort (*N* = 539)	**Overall (*N* = 933)** ^a^
**Age (years)**			
** Median (IQR)**	50.0 (43.0–58.0)	55.0 (48.0–63.0)	53.0 (45.0–61.0)
**Sex, *n* (%)**			
** Female**	386 (99.7)	537 (99.6)	930 (99.7)
** Male**	1 (0.3)	2 (0.4)	3 (0.3)
**Menopause status (female), *n* (%)**			
** Pre- or perimenopausal**	201 (52.1)	153 (28.5)	359 (38.6)
** Postmenopausal**	185 (47.9)	384 (71.5)	571 (61.4)
**BMI (kg/m^2^)**			
** Median (IQR)**	23.6 (21.6–25.7)	23.9 (21.9–26.3)	23.8 (21.6–26.1)
** Missing, *n***	38	56	95
**ECOG PS, *n* (%)**			
** 0**	213 (59.7)	171 (34.6)	388 (45.3)
** 1**	143 (40.1)	292 (59.1)	437 (51.0)
** 2**	0	23 (4.7)	23 (2.7)
** 3**	1 (0.3)	6 (1.2)	7 (0.8)
** 4**	0	2 (0.4)	2 (0.2)
** Missing, *n***	30	45	76
**ER, *n* (%)**			
** Positive**	386 (99.7)	499 (92.6)	891 (95.5)
** Negative**	1 (0.3)	3 (0.6)	4 (0.4)
** Unknown**	0	37 (6.9)	38 (4.1)
**PR, *n* (%)**			
** Positive**	353 (91.2)	412 (76.4)	771 (82.6)
** Negative**	33 (8.5)	87 (16.1)	120 (12.9)
** Unknown**	1 (0.3)	40 (7.4)	42 (4.5)
**HER2, *n* (%)** ^b^			
** Positive**	0	0	0
** Negative**	387 (100.0)	510 (94.6)	903 (96.8)
** Unknown**	0	29 (5.4)	30 (3.2)
**Stage of breast cancer, *n* (%)**			
** 0**	0	0	0
** I**	13 (3.4)	0	13 (1.4)
** II**	137 (35.4)	0	137 (14.8)
** III**	237 (61.2)	67 (12.4)	304 (32.8)
** IV**	0	472 (87.6)	472 (51.0)
** Missing, *n***	0	0	7
**Metastasis, *n* (%)**			
** Yes**	289 (74.7)	532 (98.7)	824 (88.5)
** No**	98 (25.3)	7 (1.3)	107 (11.5)
** Missing, *n***	0	0	2
**Number of metastatic sites at most recent breast cancer diagnosis, *n* (%)** ^d^			
** 1**	NA	219 (41.2)	NA
** 2**	NA	192 (36.1)	NA
** ≥3**	NA	121 (22.7)	NA
**Site(s) of distant metastasis at most recent breast cancer diagnosis, *n* (%)** ^c^ ^,^ ^d^ ^,^ ^e^			
** Any lung/bone/CNS/liver**	0	431 (81.0)	431 (52.3)
** Lung**	0	193 (36.3)	193 (23.4)
** Bone**	0	291 (54.7)	291 (35.3)
** CNS**	0	17 (3.2)	17 (2.1)
** Liver**	0	112 (21.1)	112 (13.6)

a Overall number includes 7 patients of unknown status.

b Patients with unknown status at diagnosis were verified as HER2− at initial diagnosis.

c Among patients with metastasis at breast cancer diagnosis.

d More than one option was allowed.

e Included patients with metastasis at sites other than lung, bone, CNS or liver. ABC, locally advanced or metastatic breast cancer; BMI, body mass index; CNS, central nervous system; EBC, early breast cancer; ECOG PS, Eastern Cooperative Oncology Group Performance Status; ER, estrogen receptor; HER2, human epidermal growth factor receptor-2; IQR, interquartile range; NA, not applicable; PR, progesterone receptor; SD, standard deviation.

The median age was 50.0 years (interquartile range [IQR]: 43.0–58.0) in the EBC cohort and 55.0 years (48.0–63.0) in the ABC cohort. The majority of patients were female, with three male patients enrolled (one in the EBC cohort and two in the ABC cohort). Pre- or perimenopausal status was reported in 52.1% (*n* = 201) of female patients in the EBC cohort and 28.5% (*n* = 153) in the ABC cohort. Among patients with EBC, most had stage II (35.4%, *n* = 137) or III (61.2%, *n* = 237) disease, and 74.7% (*n* = 289) had positive lymph nodes. The median (IQR) number of locally metastatic lymph nodes in the EBC cohort was 3.0 (1.0–7.0). Among patients with ABC, 36.3% (*n* = 193) had lung metastasis, 54.7% (*n* = 291) had bone metastasis, 21.1% (*n* = 112) had liver metastasis, and 3.2% (*n* = 17) had central nervous system (CNS) metastasis. Most patients with ABC had multiple metastatic sites, with 36.1% (*n* = 192) in 2 sites, and 22.7% (*n* = 121) in ≥3 sites.

### Abemaciclib exposure and treatment patterns

In the EBC cohort, 89.1% (*n* = 345) of patients received an initial dose of abemaciclib 150 mg twice daily, and 8.0% (*n* = 31) initiated with 100 mg twice daily (Table 2). Abemaciclib was received in combination with an aromatase inhibitor in 95.6% (*n* = 370) of patients.

**Table 2.** Abemaciclib treatment patterns (SAS).

	EBC cohort (*N* = 387)	ABC cohort (*N* = 539)	**Overall (*N* = 933)** ^a^
**Initial dose of abemaciclib, *n* (%)**			
** 150 mg twice daily**	345 (89.1)	444 (82.4)	796 (85.3)
** 100 mg twice daily**	31 (8.0)	80 (14.8)	111 (11.9)
** 50 mg twice daily**	2 (0.5)	3 (0.6)	5 (0.5)
** Other**	9 (2.3)	12 (2.2)	21 (2.3)
**Mean daily dose of abemaciclib (mg/day)** ^b^ ^**,**^ ^c^			
** Median (IQR)**	300.0 (300.0–300.0)	300.0 (300.0–300.0)	300.0 (300.0–300.0)
** Missing, *n***	4	9	13
**Treatment regimen,** ^d^ ***n* (%)**			
** Monotherapy**	4 (1.0)	2 (0.4)	6 (0.6)
** Combination therapy**	383 (99.0)	538 (99.8)	928 (99.5)
** Abemaciclib + fulvestrant**	6 (1.6)	205 (38.0)	213 (22.8)
** Abemaciclib + aromatase inhibitor**	370 (95.6)	326 (60.5)	701 (75.1)
** Abemaciclib + other**	9 (2.3)	11 (2.0)	20 (2.1)

a Overall number includes 7 patients of unknown status.

b Total dose of abemaciclib administration (mg) = ∑(dose[i]×frequency[i])×(number of days receiving dose[i] and frequency[i]).

c Mean daily dose of abemaciclib (mg/day) = Total dose of abemaciclib administration (mg)/∑(number of days receiving dose[i] and frequency[i]).

d Several patients were treated with more than one treatment regimen for breast cancer. ABC, locally advanced or metastatic breast cancer; EBC, early breast cancer; IQR, interquartile range; SAS, safety analysis set.

In the ABC cohort, 82.4% (*n* = 444) of patients received an initial dose of abemaciclib 150 mg twice daily, while 14.8% (*n* = 80) initiated with 100 mg twice daily (Table 2). Most patients received abemaciclib as first-line (64.7%, *n* = 349) or second-line treatment (24.7%, *n* = 133); 10.6% (*n* = 57) received abemaciclib as third- or later-line treatment. Abemaciclib was received in combination with an aromatase inhibitor in 60.5% (*n* = 326) of patients with ABC and with fulvestrant in 38.0% (*n* = 205).

### Safety

Treatment-emergent AEs (TEAEs) are summarized in Table 3. In the EBC cohort, TEAEs occurred in 85.5% (*n* = 331) of patients; 24.3% (*n* = 94) of patients experienced grade ≥3 TEAEs. The most commonly reported TEAEs in the EBC cohort were diarrhea (57.4%, *n* = 222), white blood cell (WBC) count decreased (51.2%, *n* = 198), and neutrophil count decreased (46.3%, *n* = 179). TEAEs related to abemaciclib per the investigator’s assessment occurred in 81.9% (*n* = 317) patients with EBC. SAEs occurred in 3.9% (*n* = 15) of patients. No death was considered related to abemaciclib in EBC cohort.

**Table 3.** Summary of AEs (SAS).

*n* (%)	EBC cohort (*N* = 387)	ABC cohort (*N* = 539)	**Overall (*N* = 933)** ^a^
**TEAEs**	331 (85.5)	441 (81.8)	776 (83.2)
** Treatment-related**	317 (81.9)	422 (78.3)	743 (79.6)
**Grade ≥3 TEAEs**	94 (24.3)	125 (23.2)	220 (23.6)
** Treatment-related**	80 (20.7)	102 (18.9)	183 (19.6)
**SAEs**	15 (3.9)	40 (7.4)	55 (5.9)
** Treatment-related**	5 (1.3)	17 (3.2)	22 (2.4)
**Discontinued abemaciclib due to AEs**	17 (4.4)	39 (7.2)	56 (6.0)
**Discontinued abemaciclib due to SAEs**	1 (0.3)	12 (2.2)	13 (1.4)
**Death due to AE related to treatment**	0	2 (0.4)	2 (0.2)
**TEAEs in ≥5% of patients by preferred term**			
** Diarrhea**	222 (57.4)	266 (49.4)	492 (52.7)
** WBC count decreased**	198 (51.2)	210 (39.0)	408 (43.7)
** Neutrophil count decreased**	179 (46.3)	215 (39.9)	394 (42.2)
** Anemia**	113 (29.2)	179 (33.2)	292 (31.3)
** Blood creatinine increased**	65 (16.8)	89 (16.5)	154 (16.5)
** Lymphocyte count decreased**	68 (17.6)	55 (10.2)	123 (13.2)
** Platelet count decreased**	41 (10.6)	74 (13.7)	116 (12.4)
** Hyperuricemia**	54 (14.0)	58 (10.8)	112 (12.0)
** ALT increased**	38 (9.8)	59 (10.9)	98 (10.5)
** AST increased**	35 (9.0)	53 (9.8)	89 (9.5)
** Hypertriglyceridemia**	40 (10.3)	47 (8.7)	88 (9.4)
** Decreased appetite**	20 (5.2)	60 (11.1)	80 (8.6)
** Fatigue**	17 (4.4)	48 (8.9)	65 (7.0)
** Nausea**	13 (3.4)	46 (8.5)	60 (6.4)
** GGT increased**	18 (4.7)	35 (6.5)	55 (5.9)
** Rash**	20 (5.2)	31 (5.8)	51 (5.5)

Preferred terms were coded according to MedDRA v23.1. A patient could have more than one preferred term. AEs with onset date/time ≥start date/time of abemaciclib and ≤Week 24 were collected, or at 30 days follow-up if abemaciclib discontinued before Week 24. AEs were kept in the event of a missing date.

a Overall number includes seven patients of unknown status. ABC, locally advanced or metastatic breast cancer; AE, adverse event; ALT, alanine aminotransferase; AST, aspartate aminotransferase; EBC, early breast cancer; GGT, gamma-glutamyl transferase; MedDRA, Medical Dictionary for Regulatory Activities; SAE, serious AE; SAS, safety analysis set; TEAE, treatment-emergent AE; WBC, white blood cell.

In the ABC cohort, TEAEs occurred 81.8% (*n* = 441) of patients; 23.2% (*n* = 125) experienced grade ≥3 TEAEs (Table 3). The most common TEAEs (any grade) in the ABC cohort were diarrhea (49.4%, *n* = 266), neutrophil count decreased (39.9%, *n* = 215), and WBC count decreased (39.0%, *n* = 210). TEAEs related to abemaciclib occurred in 78.3% (*n* = 422) of patients with ABC. SAEs occurred in 7.4% (*n* = 40) of patients. Two deaths were considered possibly related to abemaciclib, as judged by the investigator. One was an unexplained death in a patient with brain and multiple lung metastases who had been heavily pretreated. The patient experienced shortness of breath and chest distress/chest tightness before death with no investigations, laboratory tests, or related corrective treatment, as the patient did not go to hospital for treatment. The other was due to ILD, with the patient having had bone and liver metastases previously treated with chemotherapy and radiotherapy. The investigator judged that the relationship to treatment could not be ruled out.

AEs of special interest are summarized in Table 4. In the EBC cohort, diarrhea led to discontinuation in 2.1% (*n* = 8) patients and dose interruption or reduction in 4.4% (*n* = 17): of patients who discontinued treatment due to diarrhea, only 25.0% (*n* = 2/8) had a prior dose interruption or reduction, while 76.5% (*n* = 13/17) of patients who had dose modifications were able to continue receiving abemaciclib. Diarrhea occurred in 40.9% (*n* = 18) of patients with EBC aged ≥65 years and 59.5% (*n* = 204) of patients aged <65 years. Baseline BMI was comparable among subgroups of patients with diarrhea, without diarrhea, and in those who discontinued treatment due to diarrhea in the EBC cohort.

**Table 4.** AEs of special interest (SAS).

*n* (%)	EBC cohort (*N* = 387)	ABC cohort (*N* = 539)	**Overall (*N* = 933)** ^a^
**Diarrhea**			
** Any grade**	222 (57.4)	266 (49.4)	492 (52.7)
** Grade 1**	158 (40.8)	183 (34.0)	345 (37.0)
** Grade 2**	61 (15.8)	74 (13.7)	135 (14.5)
** Grade 3**	3 (0.8)	9 (1.7)	12 (1.3)
** Grade 4**	0	0	0
** Grade 5**	0	0	0
** SAE**	0	3 (0.6)	3 (0.3)
** Leading to discontinuation of abemaciclib**	8 (2.1)	9 (1.7)	17 (1.8)
** Leading to dose reduction**	8 (2.1)	5 (0.9)	13 (1.4)
** Leading to dose interruption**	9 (2.3)	10 (1.9)	19 (2.0)
** Leading to dose reduction/interruption**	17 (4.4)	14 (2.6)	31 (3.3)
**Neutropenia** ^b^			
** Any grade**	187 (48.3)	225 (41.7)	412 (44.2)
** Grade 1**	66 (17.1)	83 (15.4)	149 (16.0)
** Grade 2**	80 (20.7)	90 (16.7)	170 (18.2)
** Grade 3**	40 (10.3)	49 (9.1)	89 (9.5)
** Grade 4**	1 (0.3)	3 (0.6)	4 (0.4)
** Grade 5**	0	0	0
** SAE**	0	0	0
** Febrile neutropenia**	0	0	0
** Leading to discontinuation of abemaciclib**	2 (0.5)	0	2 (0.2)
** Leading to dose reduction**	7 (1.8)	4 (0.7)	11 (1.2)
** Leading to dose interruption**	13 (3.4)	16 (3.0)	29 (3.1)
** Leading to dose reduction/interruption**	20 (5.2)	19 (3.5)	39 (4.2)
**Infection**			
** Any grade**	33 (8.5)	66 (12.2)	100 (10.7)
** Grade 1**	11 (2.8)	33 (6.1)	44 (4.7)
** Grade 2**	20 (5.2)	27 (5.0)	48 (5.1)
** Grade 3**	1 (0.3)	5 (0.9)	6 (0.6)
** Grade 4**	1 (0.3)	1 (0.2)	2 (0.2)
** Grade 5**	0	0	0
** SAE**	2 (0.5)	6 (1.1)	8 (0.9)
** Leading to discontinuation of abemaciclib**	0	1 (0.2)	1 (0.1)
** Leading to dose reduction**	0	1 (0.2)	1 (0.1)
** Leading to dose interruption**	14 (3.6)	21 (3.9)	36 (3.9)
** Leading to dose reduction/interruption**	14 (3.6)	22 (4.1)	37 (4.0)
**Hepatic events**			
**ALT increased**			
** Any grade**	38 (9.8)	59 (10.9)	98 (10.5)
** Grade 1**	27 (7.0)	40 (7.4)	68 (7.3)
** Grade 2**	5 (1.3)	14 (2.6)	19 (2.0)
** Grade 3**	6 (1.6)	5 (0.9)	11 (1.2)
** Grade 4**	0	0	0
** Grade 5**	0	0	0
** SAE**	0	0	0
** Leading to discontinuation of abemaciclib**	2 (0.5)	3 (0.6)	5 (0.5)
** Leading to dose reduction**	0	0	0
** Leading to dose interruption**	7 (1.8)	1 (0.2)	8 (0.9)
** Leading to dose reduction/interruption**	7 (1.8)	1 (0.2)	8 (0.9)
**AST increased**			
** Any grade**	35 (9.0)	53 (9.8)	89 (9.5)
** Grade 1**	27 (7.0)	41 (7.6)	69 (7.4)
** Grade 2**	2 (0.5)	5 (0.9)	7 (0.8)
** Grade 3**	5 (1.3)	6 (1.1)	11 (1.2)
** Grade 4**	1 (0.3)	1 (0.2)	2 (0.2)
** Grade 5**	0	0	0
** SAE**	0	0	0
** Leading to discontinuation of abemaciclib**	1 (0.3)	3 (0.6)	4 (0.4)
** Leading to dose reduction**	0	0	0
** Leading to dose interruption**	8 (2.1)	1 (0.2)	9 (1.0)
** Leading to dose reduction/interruption**	8 (2.1)	1 (0.2)	9 (1.0)
**ILD/pneumonitis**			
** Any grade**	2 (0.5)	5 (0.9)	7 (0.8)
** Grade 1**	0	2 (0.4)	2 (0.2)
** Grade 2**	2 (0.5)	2 (0.4)	4 (0.4)
** Grade 3**	0	0	0
** Grade 4**	0	0	0
** Grade 5**	0	1 (0.2)	1 (0.1)
** SAE**	0	1 (0.2)	1 (0.1)
** Leading to discontinuation of abemaciclib**	0	1 (0.2)	1 (0.1)
** Leading to dose reduction**	0	0	0
** Leading to dose interruption**	0	0	0
** Leading to dose reduction/interruption**	0	0	0

Preferred terms were coded using MedDRA v23.1. A patient could have more than one preferred term. Severity was coded using CTCAE v5.0. The maximum severity of an event was defined as the maximum among all the severity grades reported for that particular event. Following medical review, no reported thrombotic events were considered venous thromboembolism.

a Overall number includes 7 patients of unknown status.

b Includes preferred terms: neutropenia and neutrophil count decreased. ABC, locally advanced or metastatic breast cancer; AE, adverse event; ALT, alanine aminotransferase; AST, aspartate aminotransferase; CTCAE, Common Terminology Criteria for Adverse Events; EBC, early breast cancer; ILD, interstitial lung disease; MedDRA, Medical Dictionary for Regulatory Activities; SAE, serious AE; SAS, safety analysis set.

In the ABC cohort, diarrhea led to discontinuation in 1.7% (*n* = 9) patients and dose interruption or reduction in 2.6% (*n* = 14): of patients who discontinued treatment due to diarrhea, only 22.2% (*n* = 2/9) had a prior dose interruption or reduction, while 78.6% (*n* = 11/14) of patients who had dose modifications were able to continue receiving abemaciclib. Diarrhea occurred in 38.2% (*n* = 47) of patients with ABC aged ≥65 years, and 52.6% (*n* = 219) of patients aged <65 years. Baseline BMI was comparable among subgroups of patients with diarrhea, without diarrhea and in those who discontinued treatment due to diarrhea in the ABC cohort.

The incidence of AEs among patient subgroups is summarized in Tables S1 and S2 (see online supplementary material). Incidence of TEAEs was numerically lower among the age ≥65 years subgroup compared with the <65 years subgroup (EBC, ABC), while incidence of SAEs and treatment discontinuation due to AEs were consistent between subgroups in EBC cohort. Incidence of SAEs and treatment discontinuation due to AEs were slightly higher in the age ≥65 years subgroup in ABC cohort. Incidence of TEAEs and SAEs was consistent across combination therapy (abemaciclib + aromatase inhibitor, abemaciclib + fulvestrant [ABC]) subgroups. Incidence of TEAEs, SAEs related to study treatment and grade ≥3 AEs related to study treatment, were consistent between subgroups of patients with baseline hepatic disease (*n* = 90, TEAEs 92.2%, treatment-related SAEs 1.1%, treatment-related grade ≥3 AEs 23.3%) and baseline renal impairment (*n* = 107, TEAEs 84.1%, treatment-related SAEs 2.8%, treatment-related grade ≥3 AEs 22.4%). ALT increased and AST increased occurred in 10.0% (*n* = 9) and 7.8% (*n* = 7) of patients with baseline hepatic disease, respectively, and blood creatinine increased occurred in 22.4% (*n* = 24) of patients with baseline renal impairment (all were grades 1 or 2). The most common AEs were diarrhea, WBC count decreased, neutrophil count decreased and anemia across these subgroups.

### Dose discontinuation and modification

In the EBC cohort, treatment discontinuation occurred in 10.6% (*n* = 41) of patients, most commonly due to AEs (4.4%, *n* = 17) and patient decision (4.4%, *n* = 17) (Table 5). The most common AEs leading to treatment discontinuation in the EBC cohort were diarrhea (2.1%, *n* = 8), and vomiting (0.8%, *n* = 3). Among those who discontinued treatment due to AEs, the majority of patients did not have a prior dose reduction or interruption (76.5%, *n* = 13). Dose reductions occurred in 7.0% (*n* = 27) of patients in the EBC cohort, most commonly due to AEs (5.9%, *n* = 23); WBC count decreased (2.3%, *n* = 9) and diarrhea (2.1%, *n* = 8) were the most common AEs leading to dose reduction. Among 23 patients who had a dose reduction due to AEs, 21 (91.3%) were able to stay on abemaciclib treatment during the follow-up period. Dose interruption occurred in 25.3% (*n* = 98) of patients in the EBC cohort, most commonly due to AEs (19.4%, *n* = 75); WBC count decreased was the most common AE leading to dose interruption (5.2%, *n* = 20). Among 75 patients with a dose interruption due to AEs, 70 (93.3%) were able to continue abemaciclib treatment during follow-up period.

**Table 5.** Dose adjustment (SAS).

	EBC cohort (*N* = 387)	ABC cohort (*N* = 539)	**Overall (*N* = 933)** ^a^
**Adjustment type, *n* (%)** ^b^			
***N***	146	257	405
** Discontinuation**	41 (10.6)	151 (28.0)	193 (20.7)
**Interruption**	98 (25.3)	103 (19.1)	202 (21.7)
** 1 interruption**	76 (19.6)	88 (16.3)	165 (17.7)
** 2 interruptions**	20 (5.2)	12 (2.2)	32 (3.4)
** 3 interruptions**	2 (0.5)	3 (0.6)	5 (0.5)
** Dose increased**	6 (1.6)	15 (2.8)	21 (2.3)
** Dose reduced**	27 (7.0)	41 (7.6)	68 (7.3)
** 1 dose reduction**	25 (6.5)	40 (7.4)	65 (7.0)
** 2 dose reductions**	2 (0.5)	1 (0.2)	3 (0.3)
** Change of combination type**	3 (0.8)	3 (0.6)	6 (0.6)
**Patients with any AE leading to treatment discontinuation, (>0.3% patients overall), *n* (%)**			
***n***	17 (4.4)	39 (7.2)	56 (6.0)
** Diarrhea**	8 (2.1)	9 (1.7)	17 (1.8)
** Vomiting**	3 (0.8)	3 (0.6)	6 (0.6)
** ALT increased**	2 (0.5)	3 (0.6)	5 (0.5)
** AST increased**	1 (0.3)	3 (0.6)	4 (0.4)
** WBC count decreased**	1 (0.3)	3 (0.6)	4 (0.4)
**Patients with any AE leading to dose reduction, (>0.3% patients overall), *n* (%)**			
***n***	23 (5.9)	26 (4.8)	49 (5.3)
** WBC count decreased**	9 (2.3)	6 (1.1)	15 (1.6)
** Diarrhea**	8 (2.1)	5 (0.9)	13 (1.4)
** Neutrophil count decreased**	7 (1.8)	4 (0.7)	11 (1.2)
** Decreased appetite**	0	5 (0.9)	5 (0.5)
** Vomiting**	3 (0.8)	2 (0.4)	5 (0.5)
** Anemia**	1 (0.3)	3 (0.6)	4 (0.4)
**Patients with any AE leading to dose interruption, (>1% patients overall), *n* (%)**			
***n***	75 (19.4)	84 (15.6)	160 (17.1)
** WBC count decreased**	20 (5.2)	24 (4.5)	44 (4.7)
** Neutrophil count decreased**	13 (3.4)	16 (3.0)	29 (3.1)
** Diarrhea**	9 (2.3)	10 (1.9)	19 (2.0)
** COVID-19**	9 (2.3)	9 (1.7)	18 (1.9)
** Platelet count decreased**	2 (0.5)	8 (1.5)	10 (1.1)

Preferred terms were coded using MedDRA v23.1. A patient could have more than one preferred term.

a Overall number includes 7 patients of unknown status.

b A patient could have ≥2 adjustment types if they had ≥2 dose changes during the study period, and a reason was counted only once if they had dose changes more than once due to the same reason. ABC, locally advanced or metastatic breast cancer; AE, adverse event; ALT, alanine aminotransferase; AST, aspartate aminotransferase; COVID-19, coronavirus disease 2019; EBC, early breast cancer; MedDRA, Medical Dictionary for Regulatory Activities; SAS, safety analysis set; WBC, white blood cell.

In the ABC cohort, abemaciclib was discontinued in 28.0% (*n* = 151) of patients, most commonly due to disease progression (10.4%, *n* = 56), and 7.2% (*n* = 39) of patients had AEs leading to treatment discontinuation (Table 5). The most common AEs leading to treatment discontinuation in the ABC cohort were diarrhea (1.7%, *n* = 9), followed by vomiting, ALT increased, AST increased, and WBC count decreased (each 0.6%, *n* = 3). Among those who discontinued treatment due to AEs, the majority did not have a prior dose reduction or interruption (76.9%, *n* = 30). Dose reductions occurred in 7.6% (*n* = 41) of patients with ABC, most commonly due to AEs (4.8%, *n* = 26); WBC count decreased was the most common AE leading to dose reduction (1.1%, *n* = 6). Among 26 patients who had a dose reduction due to AEs, 22 (84.6%) were able to stay on abemaciclib treatment during follow-up. Dose interruption occurred in 19.1% (*n* = 103) of patients in the ABC cohort, most commonly due to AEs (15.6%, *n* = 84): WBC count decreased was the most common AE leading to dose interruption (4.5%, *n* = 24). Among the patients with dose interruption due to AEs, 71 (84.5%) were able to continue receiving abemaciclib during the follow-up period.

### Effectiveness

In the EBC cohort, the Week 24 EFS rate was 99.7% (*N* = 302, 95% CI: 97.6–100.0; Figure S2—see online supplementary material for a color version of this figure), with results comparable between age subgroups, and regardless of dose reduction (Figure S3—see online supplementary material for a color version of this figure). In the ABC cohort, the Week 24 EFS rate was 85.1% (*N* = 468, 95% CI: 81.3–88.2); among patients receiving first-line, second-line, or ≥third-line abemaciclib treatment, Week 24 EFS rates were 91.2% (*n* = 302), 79.0% (*n* = 118), and 62.5% (*n* = 48), respectively. EFS rates were comparable across age groups, irrespective of combination partner (aromatase inhibitor or fulvestrant) in first-line therapy in the ABC cohort. Clinical benefit was maintained when patients had their dose reduced due to AEs (Figure S3—see online supplementary material for a color version of this figure).

## Discussion

As the largest prospective observational study of abemaciclib treatment in China to date, this study describes the real-world safety and effectiveness profile of abemaciclib in patients with HR+/HER2− breast cancer in China. This study enrolled over 900 patients from 32 hospitals across China, applying broader inclusion criteria compared to the stringent eligibility requirements of the MONARCH series clinical trials.^13^^,^^15^^,^^18^^,^^21^ The study population may therefore more accurately represent the real-world epidemiology of breast cancer in China and enable prescribers to relate these results to real-world clinical practice, in which abemaciclib is a widely used option of the first four CDK4/6 inhibitors approved in China.^22^^,^^23^

Baseline demographics, disease characteristics, and treatment patterns of patients receiving abemaciclib enrolled in the present study were generally comparable with the MONARCH trials. For example, patients in the EBC cohort had a median of 3.0 (IQR: 1.0–7.0) positive lymph nodes at most recent breast cancer diagnosis, a requirement of the monarchE study (≥4 locally metastatic lymph nodes or 1–3 nodes with tumor size ≥5 cm, histologic grade 3, or Ki-67 ≥ 20%). Moreover, the majority of patients in the ABC cohort received abemaciclib in the first-line (64.7%, *n* = 349) or second-line (24.7%, *n* = 133), as in the MONARCH 2, 3, and MONARCH plus trials.^13^^,^^15^^,^^21^ However, the present study included a broader population of patients with breast cancer than in clinical trials to date, including patients with an Eastern Cooperative Oncology Group Performance Status (ECOG PS) score of 2–4, pre-/peri-menopausal women with ABC, as well as those receiving third- or later-line abemaciclib and with CNS metastasis associated with poorer prognoses. Therefore, the patient population included in this study may better represent the population characteristics of breast cancer in China than clinical trials to date.

Incidences of AEs (including the three most common TEAEs of diarrhea, WBC count decreased, and neutrophil count decreased), SAEs, and treatment discontinuation following abemaciclib administration were generally comparable or slightly lower than that reported in clinical trials in both EBC and ABC cohorts.^13–15^^,^^17^^,^^18^ TEAEs were reported in 331 (85.5%) patients with EBC and 441 (81.8%) with ABC, the majority of which were grades 1 or 2 in severity, while incidence ranged from 97.9% to 100% in the MONARCH series trials.^13–15^^,^^17^^,^^18^ Incidence of SAEs (3.9% in EBC and 7.4% in ABC) was also comparatively lower than that reported in the MONARCH trials (range: 12.3–27.5%); the incidence of abemaciclib discontinuation due to AEs (4.4% in EBC and 7.2% in ABC in the present study) was within the 3.9–25.1% range in the MONARCH trials.^13–15^^,^^17^^,^^18^^,^^21^^,^^24^

In the EBC cohort, the incidence of TEAEs was numerically lower among patients aged ≥65 years compared with those aged <65 years, while the incidence of SAEs and discontinuation due to AEs was comparable between age subgroups. Notably in the ABC cohort, the incidence of TEAEs was also lower among patients aged ≥65 years with a slightly higher incidence of SAEs and discontinuation rate. While this differs from the higher incidence of TEAEs following abemaciclib treatment observed among older patients reported previously,^25^^,^^26^ the self-reported burden of AEs was lower among those aged ≥65 years compared with those aged <65 years in a long-term follow-up of the monarchE study.^27^ Therefore, this observation may potentially be explained by a poorer perception or less frequent reporting of low-grade AEs among older patients.^27^ Incidence of TEAEs and SAEs was consistent across combination therapy (abemaciclib + aromatase inhibitor, abemaciclib + fulvestrant) subgroups in the ABC cohort. The safety profile of abemaciclib in patients with baseline hepatic disease or renal impairment was generally consistent with the overall population, with no new safety signals identified.

In the present study, the majority of patients initiated abemaciclib treatment with the recommended full dose (150 mg twice daily). Most patients were able to continue receiving abemaciclib treatment with dose modification while deriving clinical benefit. In patients who discontinued abemaciclib due to AEs, including diarrhea, the majority did not attempt prior dose reduction or interruption, while among those who had a dose reduction or interruption due to AEs, the majority were able to stay on abemaciclib treatment. Similar trends in dose modification and discontinuation were observed in a real-world population of US patients in a recent retrospective analysis.^28^ Among 160 patients with dose interruption due to AEs overall, 142 (88.8%) continued to receive abemaciclib, and among 49 patients with dose reduction due to AEs, 43 (87.8%) remained on abemaciclib. These findings were consistent across both EBC and ABC patient cohorts. These results indicate that in the real-world setting, full-dose initiation of abemaciclib according to the label is relatively well tolerated, and dose modifications may help patients stay on treatment without affecting treatment effectiveness; indeed, a recent analysis of the monarchE study showed that IDFS rates were not compromised by dose reductions among patients with EBC.^29^ Patients should therefore be counseled about symptom management and monitored closely for AEs, with dose modification offered to patients as a management strategy to improve tolerability without impacting effectiveness, prior to permanent treatment discontinuation.

The EFS rate at Week 24 was 85.1% in the ABC cohort, with subgroup results consistent across age, treatment combination, and dose modification. These results support the use of abemaciclib treatment in patients aged ≥65 years and show a comparable effectiveness profile regardless of treatment combination partner and dose modification. Effectiveness results were generally consistent with results reported in clinical trials.^13–15^

Limitations of this study include those inherent to observational studies, including potential bias in patient selection; to minimize this risk, all patients meeting study criteria were invited to participate. While the combined safety data from both EBC and ABC patient cohorts are useful to characterize the overall real-world safety and tolerability profiles of abemaciclib, these combined data need to be interpreted with some caution, since the presentation of AEs and patient treatment goals may differ between early and advanced settings. In addition, seven patients with unknown disease stages were included in the overall population of 933 patients in the SAS; however, due to the small proportion, this was not expected to have a meaningful impact on the results or conclusions, and analyses of EBC and ABC cohorts are reported separately. Underreporting of AEs may also have introduced bias in data recording, owing to the observational nature of the study: AEs lacking signs and symptoms in the early stages that may only be detected by routine laboratory monitoring may be underestimated in a real-world setting.^30^ Moreover, owing to the 6-month safety follow-up, safety events that required long-term exposure or that had a long latency period could not be captured in the present study. The 6-month duration of follow-up at this analysis also precluded longer term estimation of EFS beyond Week 24; however, follow-up for EFS and OS (up to two years) is ongoing. The frequency of tumor evaluations was performed per routine clinical practice, which may have been less frequent than predefined schedules of clinical trials, potentially leading to overestimation of EFS.

## Conclusions

The real-world safety, tolerability, and effectiveness profiles of abemaciclib in Chinese patients with HR+/HER2– EBC and ABC were consistent with results of previous clinical trials, with no new safety signals identified.^13–15^^,^^17^^,^^18^ The majority of patients who discontinued abemaciclib treatment did not attempt dose reduction or interruption, while most patients whose dose was modified were able to continue treatment, suggesting dose modification should be considered to improve treatment tolerability. These results confirm that the benefit-risk profile of abemaciclib is positive in the real-world clinical setting in China.

## Supplementary Material

oyag013_Supplementary_Data

## Data Availability

De-identified data underlying this work are available from the corresponding author on reasonable request.
