# Supplementary material for: A prospective post-marketing observational study of abemaciclib in patients with HR+, HER2− breast cancer in China
Source: Oncologist. 2026 Jan 23;31(4):oyag013. doi: 10.1093/oncolo/oyag013 (PMC12978423; doi:10.1093/oncolo/oyag013)
Supplement: oyag013_Supplementary_Data [file oyag013_supplementary_data.docx]

# Supplementary Appendix

This appendix has been provided by the authors to give additional information about their work.

Supplement to:

**A prospective post-marketing observational study of abemaciclib in patients with HR+, HER2− breast cancer in China**

Quchang Ouyang^1,†^, Peng Yuan^2,†^, Jianxia Liu^3^, Yuee Teng^4^, Zhihua Li^5^, Xuening Ji^6^, Lina Liu^7^, Mopei Wang^8^, Liqun Zou^9^, Ling Xu^10^, Suisheng Yang^11^, Zhenxin Zhu^12^, Liu Yang^12^, Jinnan Li^12^, Qiang Liu^13,^*

^1^Hunan Cancer Hospital, Changsha, China; ^2^National Cancer Center / National Clinical Research Center for Cancer / Cancer Hospital, Chinese Academy of Medical Sciences and Peking Union Medical College, Beijing, China; ^3^The First Affiliated Hospital of Soochow University, Suzhou, China; ^4^The First Affiliated Hospital of China Medical University, Shenyang, China; ^5^Nanchang People’s Hospital, Nanchang, China; ^6^Zhongshan Hospital Affiliated to Dalian University, Dalian, China; ^7^Nanyang Second General Hospital, Nanyang, China; ^8^Peking University Third Hospital, Beijing, China; ^9^West China Hospital of Sichuan University, Chengdu, China; ^10^Peking University First Hospital, Beijing, China; ^11^Gansu Cancer Hospital, Lanzhou, China; ^12^Eli Lilly and Company, Shanghai, China; ^13^Sun Yat-sen Memorial Hospital, Sun Yat-sen University, Guangzhou, China

^†^These authors contributed equally to this work.

*Corresponding author

## Supplemental Methods

### *Classification of breast cancer status*

- For patients with a current diagnosis of stage 0/I/II breast cancer, patients were classified as having early breast cancer (EBC).
- For patients with a current diagnosis of stage IV breast cancer, patients were classified as having locally advanced or metastatic breast cancer (ABC).
- For patients with a current diagnosis of stage III breast cancer, if they had received prior systemic therapy in the advanced setting, then they were classified as having ABC. If the patient had no prior systemic therapy in the advanced setting, then the radical surgery history for breast cancer (± 7 months of the date of current diagnosis) was referred to. If the patient had received no radical surgery for breast cancer, then they were classified as having ABC; if they had received radical surgery for breast cancer, then they were classified as having EBC.

## Supplemental Table S1. Incidence of AEs by patient age (SAS)

| **n (%)** | **EBC cohort  (N=387)** | **ABC cohort  (N=539)** | **Overall   (N=933)^a^** |
| --- | --- | --- | --- |
| **Age** |  |  |  |
| **<65 years, N** | 343 | 416 | 765 |
| TEAEs | 298 (86.9) | 344 (82.7) | 646 (84.4) |
| Grade ≥3 | 86 (25.1) | 95 (22.8) | 182 (23.8) |
| SAEs | 12 (3.5) | 23 (5.5) | 35 (4.6) |
| Treatment-related | 4 (1.2) | 10 (2.4) | 14 (1.8) |
| Discontinued abemaciclib due to TEAEs | 15 (4.4) | 26 (6.3) | 41 (5.4) |
| Discontinued abemaciclib due to SAEs | 0 | 9 (2.2) | 9 (1.2) |
| **≥65 years, N** | 44 | 123 | 168 |
| TEAEs | 33 (75.0) | 97 (78.9) | 130 (77.4) |
| Grade ≥3 | 8 (18.2) | 30 (24.4) | 38 (22.6) |
| SAEs | 3 (6.8) | 17 (13.8) | 20 (11.9) |
| Treatment-related | 1 (2.3) | 7 (5.7) | 8 (4.8) |
| Discontinued abemaciclib due to TEAEs | 2 (4.6) | 13 (10.6) | 15 (8.9) |
| Discontinued abemaciclib due to SAEs | 1 (2.3) | 3 (2.4) | 4 (2.4) |

^a^Overall number includes 7 patients of unknown status. ABC, locally advanced or metastatic breast cancer; AE, adverse event; EBC, early breast cancer; SAE, serious AE; SAS, safety analysis set; TEAE, treatment-emergent AE.

## Supplemental Table S2. Incidence of AEs by treatment combination partner in the ABC cohort (SAS)

| **n (%)** | **ABC cohort  (N=539)** |
| --- | --- |
| **Combination therapy** |  |
| **Abemaciclib + aromatase inhibitor, N** | 322 |
| TEAEs | 268 (83.2) |
| Grade ≥3 | 72 (22.4) |
| SAEs | 27 (8.4) |
| Treatment-related | 14 (4.4) |
| Discontinued abemaciclib due to TEAEs | 25 (7.8) |
| Discontinued abemaciclib due to SAEs | 10 (3.1) |
| **Abemaciclib + fulvestrant, N** | 203 |
| TEAEs | 160 (78.8) |
| Grade ≥3 | 50 (24.6) |
| SAEs | 12 (5.9) |
| Treatment-related | 2 (1.0) |
| Discontinued abemaciclib due to TEAEs | 14 (6.9) |
| Discontinued abemaciclib due to SAEs | 2 (1.0) |

ABC, locally advanced or metastatic breast cancer; AE, adverse event; SAE, serious AE; SAS, safety analysis set; TEAE, treatment-emergent AE.


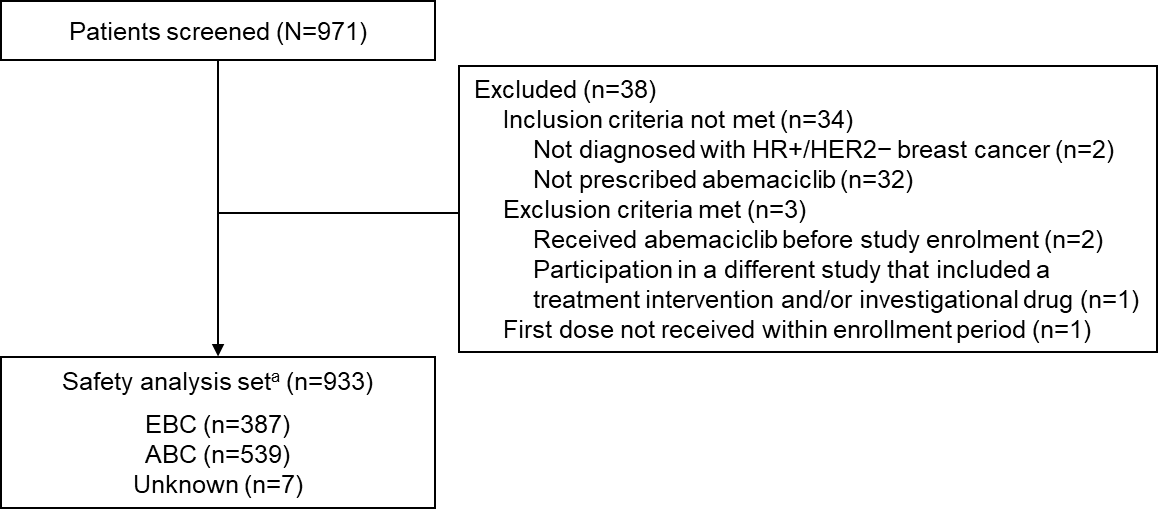


## Supplemental Figure S1. Patient flow chart

^a^The safety analysis set included all patients who took at least one dose of abemaciclib. ABC, locally advanced or metastatic breast cancer; EBC, early breast cancer; HER2, human epidermal growth factor receptor-2; HR, hormone receptor.

**
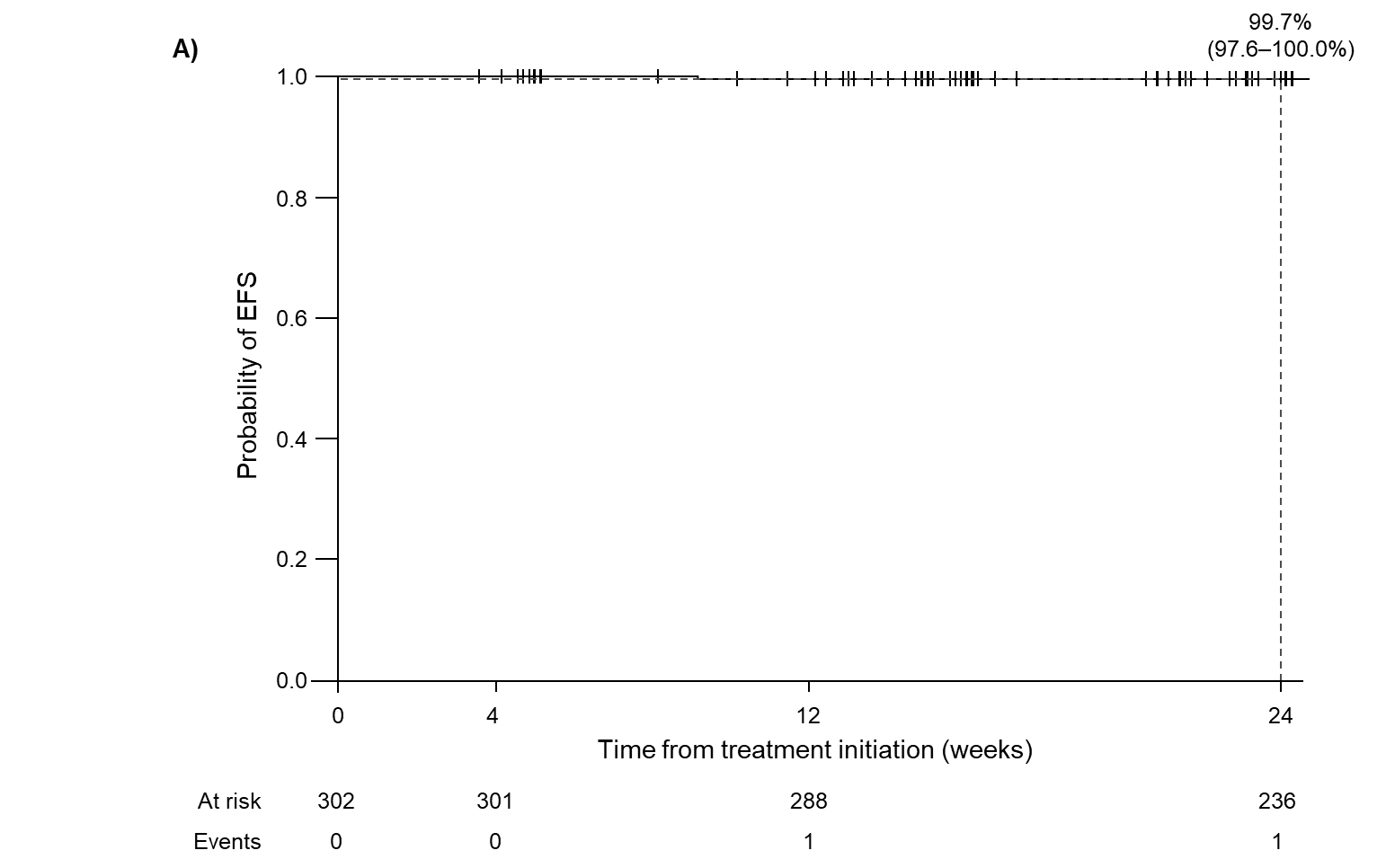
**

**
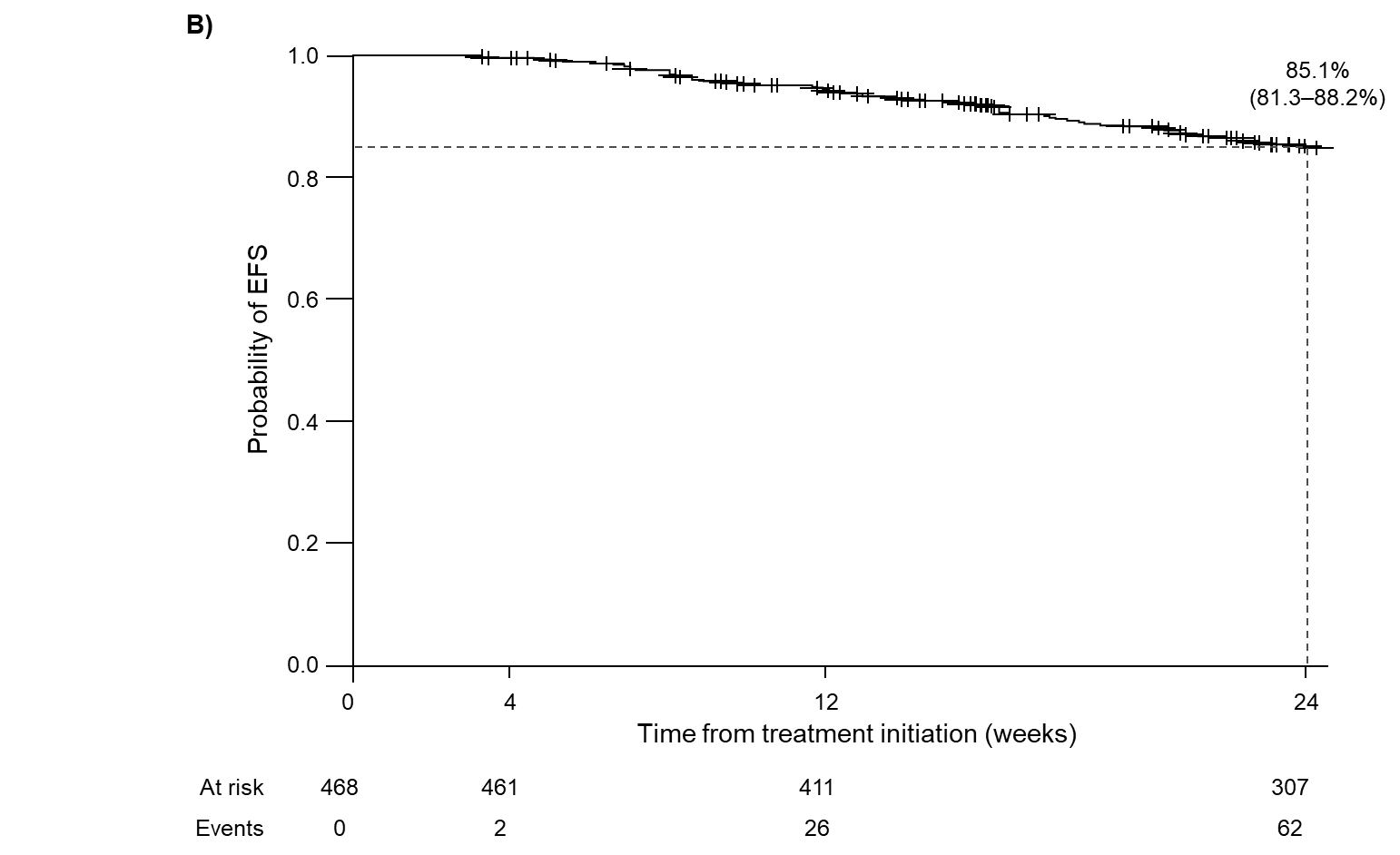
**

## Supplemental Figure S2. Kaplan-Meier plots for EFS in patients receiving abemaciclib treatment in the EBC (A), and ABC (B) cohorts

Population included all patients who took at least one dose of abemaciclib and had a baseline and any postbaseline follow up EFS data. Patients with EBC who received neoadjuvant abemaciclib were excluded from the EBC category in this analysis. ABC, locally advanced or metastatic breast cancer; EBC, early breast cancer; EFS, event-free survival.


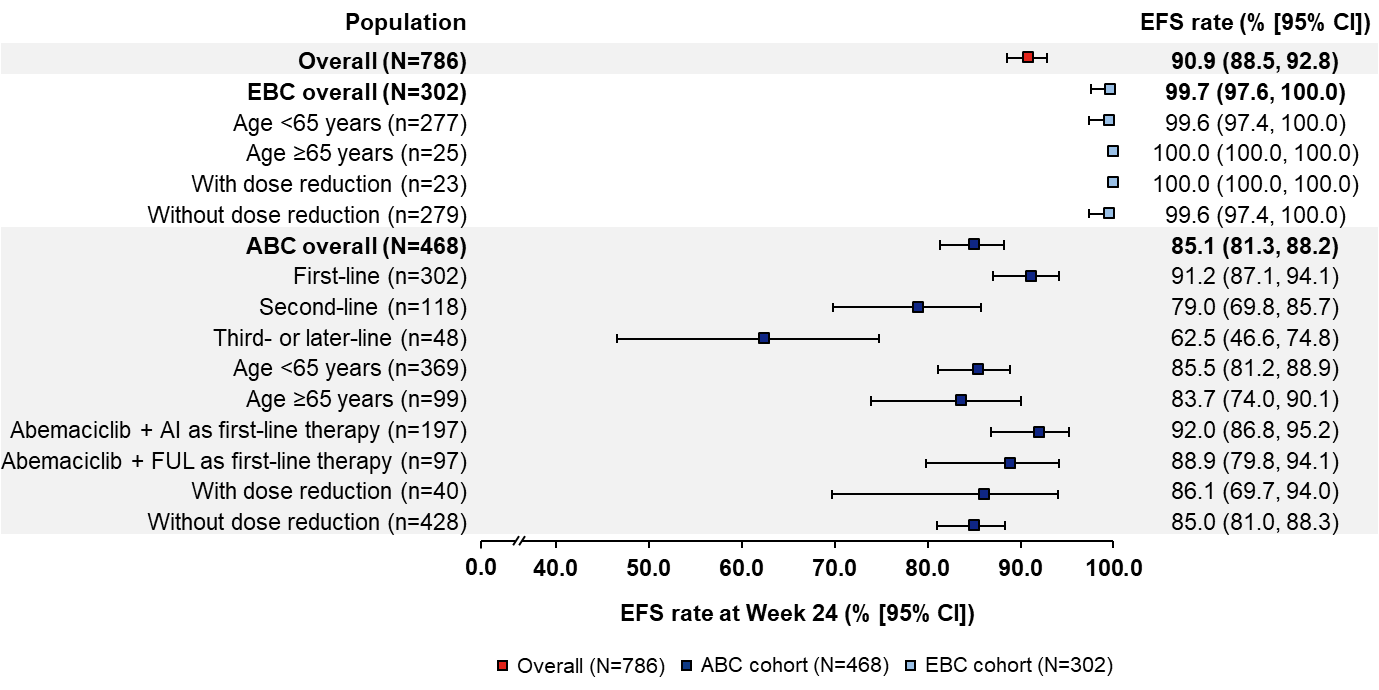


## Supplemental Figure S3. EFS in subgroups of patients receiving abemaciclib treatment at Week 24

Population included all patients who took at least one dose of abemaciclib and had a baseline and any postbaseline follow up EFS data. Patients with EBC who received neoadjuvant abemaciclib were excluded from the EBC category in this analysis. ABC, locally advanced or metastatic breast cancer; AI, aromatase inhibitor; CI, confidence interval; EBC, early breast cancer; EFS, event-free survival; FUL, fulvestrant.
